# Impact of movement training on upper limb motor strategies in persons with shoulder impingement syndrome

**DOI:** 10.1186/1758-2555-1-8

**Published:** 2009-05-17

**Authors:** Jean-Sébastien Roy, Hélène Moffet, Bradford J McFadyen, Richard Lirette

**Affiliations:** 1 Centre for Interdisciplinary Research in Rehabilitation and Social Integration, Quebec City, Canada; 2 Department of Rehabilitation, Faculty of Medicine, Laval University, Quebec City, Canada; 3 Club Entrain Medical Centre, Quebec City, Canada

## Abstract

**Background:**

Movement deficits, such as changes in the magnitude of scapulohumeral and scapulathoracic muscle activations or perturbations in the kinematics of the glenohumeral, sternoclavicular and scapulothoracic joints, have been observed in people with shoulder impingement syndrome. Movement training has been suggested as a mean to contribute to the improvement of the motor performance in persons with musculoskeletal impairments. However, the impact of movement training on the movement deficits of persons with shoulder impingement syndrome is still unknown. The aim of this study was to evaluate the short-term effects of supervised movement training with feedback on the motor strategies of persons with shoulder impingement syndrome.

**Methods:**

Thirty-three subjects with shoulder impingement were recruited. They were involved in two visits, one day apart. During the first visit, supervised movement training with feedback was performed. The upper limb motor strategies were evaluated before, during, immediately after and 24 hours after movement training. They were characterized during reaching movements in the frontal plane by EMG activity of seven shoulder muscles and total excursion and final position of the wrist, elbow, shoulder, clavicle and trunk. Movement training consisted of reaching movements performed under the supervision of a physiotherapist who gave feedback aimed at restoring shoulder movements. One-way repeated measures ANOVAs were run to analyze the effect of movement training.

**Results:**

During, immediately after and 24 hours after movement training with feedback, the EMG activity was significantly decreased compared to the baseline level. For the kinematics, total joint excursion of the trunk and final joint position of the trunk, shoulder and clavicle were significantly improved during and immediately after training compared to baseline. Twenty-four hours after supervised movement training, the kinematics of trunk, shoulder and clavicle were back to the baseline level.

**Conclusion:**

Movement training with feedback brought changes in motor strategies and improved temporarily some aspects of the kinematics. However, one training session was not enough to bring permanent improvement in the kinematic patterns. These results demonstrate the potential of movement training in the rehabilitation of movement deficits associated with shoulder impingement syndrome.

## Background

Movement deficits have been observed in persons with musculoskeletal disorders [1-3]. A cortical reorganization consecutive to peripheral impairments may explain such deficits [4-6]. Interestingly, it has been demonstrated that movement training can induce change in the cortical organization of healthy subjects [7] and contribute to the improvement of the motor performance in persons with peripheral impairments [2,8]. In order to efficiently rehabilitate the deficits, movement training should, however, be based on the best strategies available to favour motor learning. Factors such as the use of instruction, demonstration and extrinsic feedback during movement training have been proved to promote motor learning [9-11]. Among them, extrinsic feedback is one of the most potent factors [10]. Extrinsic feedback is given by an external source and provides error information that can be used in addition to the person's own intrinsic error signals [9]. According to Fitts & Posner [12], the first phase of learning is the cognitive stage where one has to solve the problem and decide what to do. It is during this stage that the use of extrinsic feedback is thought to be the most effective since it brings awareness to movement deficits [11].

Shoulder impingement syndrome (SIS) has been described as a repeated mechanical compression of the subacromial structures under the coracoacromial arch during arm elevation [13]. Studies suggest that persons with SIS may benefit from movement training with extrinsic feedback. Indeed, it was shown that they present movement deficits during arm elevation. These deficits range from changes in the magnitude of scapulohumeral and scapulathoracic muscle activations [14-16], to perturbations in the kinematics of the scapula (increased or decreased scapular posterior tilting and lateral rotation) [14,17,18], clavicle (increased elevation and retraction) [18,19] and humeral head (superior displacement with respect of the glenoid) [20] during arm movement. These deficits most likely contribute to impingement of the subacromial structures and subsequent pain during arm movement.

The nature and importance of these deficits differ among persons with SIS [18,19,21]. It has been shown that half of the persons with SIS used a different motor strategy compared to a control group during reaching tasks in the frontal plane [19]. Specifically, these persons used more trunk rotation and clavicular elevation, and finished reaching with the trunk more rotated, clavicle more elevated and shoulder in a more anterior plane of elevation. The explanation for these results is that such movement strategies may be used to protect the impaired shoulder following superior displacement of the humeral head during arm elevation [19,20]. By using more trunk rotation, persons with SIS elevate their arm in a manner that prevents them from going into the frontal plane where the subacromial space is minimal [22]. Furthermore, by elevating their clavicle, they can reach the target even though the humeral head is superiorly migrated. It suggests that at least a portion of the persons with SIS present deficits that could be rehabilitated by movement training. Reduction of these impairments could be an important factor in reaching a normal level of shoulder function.

It is still unknown how the motor strategies of persons with SIS are influenced by movement training. In fact, the effects of movement training have never been evaluated for persons with SIS on variables related to motor control, such as muscular activation or kinematic patterns. The aim of this study was to evaluate the immediate and short-term effects of movement training with extrinsic feedback on the motor strategies of persons with SIS using such variables (muscular activation and kinematic patterns). A second aim was to determine how subgroups of persons with SIS who present significant or slight motor deficits respond to the training. We think that movement training will help reduce the movement deficits of persons with shoulder impingement syndrome.

## Methods

### Participants

Thirty-three subjects with SIS, diagnosed by an orthopaedic surgeon, were recruited (Table 1). They were included if they had at least one positive finding in each of the following categories [19]: 1) painful arc of movement during flexion or abduction; 2) positive Neer or Kennedy-Hawkins impingement signs; and 3) pain on resisted lateral rotation, abduction or Jobe test. The exclusion criteria were: type III acromion; calcification; shoulder instability; previous shoulder surgery; and shoulder pain reproduced during neck movement. A control group composed of 20 subjects with no shoulder pathology was also recruited. All subjects provided informed consent. This study was approved by the Ethics Committee of the Quebec Rehabilitation Institute.

**Table 1:** Subjects' characteristics (Mean ± 1 standard deviation or n (%))

		SIS subjects		
		
Variables	Control group (n = 20)	SIS group (n = 33)	SISele subgroup (n = 17)	SISdep subgroup (n = 10)
Age (y)	46.6 ± 9.9	47.9 ± 8.7	48.4 ± 9.6	45.2 ± 9.2
Gender: Women	13 (65.0%)	22 (66.7%)	12 (70.6%)	6 (60%)
Right hand dominance	17 (85.0%)	28 (84.8%)	15 (88.2%)	8 (80.0%)
Dominant side evaluated	12 (60.0%)	21 (63.6%)	13 (76.5%)	6 (60.0%)
Disease duration (months)		10.8 ± 9.0	11.7 ± 10.8	9.9 ± 6.7
DASH score (0–100)		33.3 ± 12.1	35.9 ± 12.5	27.9 ± 8.9

### Study design

Subjects with SIS were involved in two visits, one day apart. The motor strategies of the upper limb were measured at each of the four evaluation phases of the study: E1) before (baseline), E2) during, E3) immediately after, and, E4) 24 hours after movement training with feedback. At the first visit, prior to the measurement of the motor strategies, an established self-reported questionnaire, the Disabilities of the Arm, Shoulder and Hand (DASH) questionnaire, was completed to assess upper limb pain and functional level [23]. Thereafter, baseline motor strategies were evaluated during reaching movements. This baseline evaluation (E1) was followed by an education period on shoulder anatomy and on specific deficits related to impingement using an anatomical model of the shoulder. Then, motor strategies during reaching were re-evaluated during (E2) and immediately after (E3) movement training with feedback. The day after, subjects came back for the second visit where motor strategies during reaching were re-evaluated (E4). Subjects in the control group only performed the reaching tasks once during a visit in order to assess normal motor strategies.

### Measurement of motor strategies during reaching

#### Reaching tasks

The tasks consisted of reaching out and pointing (with contact) to targets located in two planes of elevation. Subjects were asked to execute reaching at a natural speed, as if they were performing activities of daily living. In each plane, 10 trials of reaching movement were performed. Using the Present Pain Index, pain level was evaluated after each trial. The symptomatic arm was evaluated for the SIS group. For the control group, the side was chosen to have the same proportion of dominant/non-dominant sides as evaluated in the SIS group. For the trials before, immediately after and 24 hours after movement training, a random sequence of trials was established. During movement training, all trials in a plane of movement were first carried out before performing the other movement plane and the first plane of movement executed was balanced between subjects in order to have half the subjects starting in each plane. In a seated position, reaching tasks started with the upper limb in a neutral position at the side of the body and the tip of the second finger in contact with a pressure switch. One target was located in the frontal plane and positioned at a distance equivalent to the subject's arm length and at a height equivalent to the position of the second finger when the shoulder was at 90° of abduction. The other target was positioned at the same length and height as the target in the frontal plane, but located in front of the contralateral foot, in a sagittal/oblique plane between flexion and horizontal adduction. Pressure switches were placed under each target to signal the end of reaching. Motor strategies were defined by reaching speed, upper limb kinematic patterns of relative joint angles and electromyographic (EMG) activity of seven muscles. Based on previous findings that have shown only slight deficits for subjects with SIS during reaching in the sagittal/oblique plane [19], only the frontal plane data are presented here.

#### Kinematic

Kinematic data were recorded using the Optotrak system (Northern Digital Inc, 103 Randall Drive, Waterloo, Ontario, Canada N2V 1C5). Triads of infrared light-emitting diodes were positioned on the hand (dorsal face), forearm (proximal to the styloid process of the radius), upper-arm (near the insertion of the deltoid), clavicle (lateral part of the clavicle) and trunk (top of the sternum). Data were sampled at 100 Hz and digitally low-pass filtered at 8 Hz. Fourteen bony landmarks were digitized before the acquisition of data in order to recreate the local coordinate systems which, along with joint rotations, were defined according to the International Society of Biomechanics recommendations [24]. To compare the four evaluation phases, two periods (auditory cue to beginning of the movement; beginning of the movement to end of the movement) of 100 points each were defined. Each point represented 1% of each period. Movement amplitudes were plotted for the wrist (hand relative to forearm: flex/extension; radial/ulnar deviation), elbow (forearm relative to arm: flex/extension), shoulder (humerus relative to trunk: plane of elevation; elevation; rotation), S/C joints (clavicle relative to trunk: retraction/protraction; elevation/depression) and trunk (trunk relative to global system: flex/extension; rotation; lateral flexion). Thereafter, joint position at the end of reaching, as well as total joint excursion (absolute value of the difference between maximum and minimum amplitude that occurred during reaching) were calculated. Maximal hand speed was also calculated.

#### Electromyography

Bipolar surface EMG electrodes were used to record the muscular activity of the upper, middle and lower trapezius, serratus anterior, infraspinatus, and anterior and middle deltoid. Following skin preparation, Ag/AgCl electrodes (Kendall Medi-Trace 100, Tyco Healthcare Group, Mansfield, MA 02048) were placed over the muscle belly, parallel to the direction of the muscle fibres [25]. A reference electrode was placed over the contralateral acromion. Verification of the electrode placement and EMG signal quality was completed by visual monitoring of EMG signals while subject performed a voluntary contraction [25]. Raw EMG signals were amplified for a total gain of 4000 and transmitted by optical fibre to a multichannel Neogenix main receiver (NEO 210A, Neogenix Technologies, 100–3175 Quatre-Bourgeois, Quebec City, Quebec, Canada G1W 2K7). The EMG signal was band pass-filtered at 10–500 Hz, converted from analog to digital (1000 Hz) and stored. Using specially developed software, EMG signals were filtered with a digital high-pass Butterworth filter at a frequency of 10 Hz to minimize the effect of movement artefacts and full-wave rectified. EMG activity was normalized to a reference condition and normalized results were expressed as a percent of the reference condition. The reference condition, recorded before the experiment, consisted in the mean EMG activity while the subject maintained his arm at the target position with a load of 1 kg in his hand for five seconds. Mean normalized EMG activity was calculated for three phases: pre-movement (beginning of muscle activation to beginning of movement), acceleration (beginning of movement to the end of the hand acceleration) and deceleration (beginning of hand deceleration to the end of the movement).

### Movement training with extrinsic feedback

Movement training was performed under the supervision of a physiotherapist who gave feedback aimed at correcting shoulder girdle movement [26]. The results of the trials performed before supervised training were used to determine the feedback given during supervised training. Therefore, the type of feedback was established according to individualized impairments. Three types of feedback were given: visual, using a mirror; manual, by restricting shoulder girdle movements or guiding scapular movements; and verbal, with comments related to the motor performance. Manual and verbal feedbacks were standardized for each type of altered shoulder kinematics [26]. In each elevation plane, a trial with feedback was followed by a trial without feedback for a total of 10 trials in each plane. During the trials with feedback, subjects first executed the reaching movement with the unimpaired side in front of a mirror. Then, still in front of a mirror, they executed the same movement with the impaired side. During movement, subjects received manual feedback if the kinematic of the shoulder was altered. Following the movement with feedback, they had to evaluate their own performance, and finally, they received verbal feedback related to the motor aspect of the movement that had to be improved for the next trial (example: elevation of your shoulder girdle was too important). If the movement was adequate, the verbal feedback confirmed that the movement was properly executed. No other exercises or training were performed during this visit.

### Statistical analysis

Mean value of the 10 trials during reaching in the frontal plane was used for the statistical analysis. First, the baseline motor strategies (total joint excursion, final joint position and normalized EMG activity) of the SIS group were compared the ones of the control group using independent t-tests. Then, for the SIS group, the effect of movement training was analysed using one-way (shoulder pain, reaching speed and kinematic patterns [total joint excursion and final joint position]) and two-way (normalized EMG activity) repeated measures ANOVA. The factors in the model were the evaluation phase (before [E1], during [E2], immediately after [E3] and the day after training with feedback [E4]) and, for the normalized EMG activity, the reaching phases (pre-movement, acceleration and deceleration). Paired t-tests, with Bonferroni adjustment, were used for multiple pairwise comparisons. Multiple pairwise comparisons were only performed compared to the baseline trials (E1 vs. E2; E1 vs. E3; E1 vs. E4). Data of the control group were not used for the evaluation of the effects of movement training. All analyses were conducted with the SPSS software (Version 12; SPSS Inc, 233 S Wacker Dr, 11th Fl, Chicago, Illinois 60606, USA). The alpha level was set at 0.05.

Two SIS subgroups were also defined at baseline according to the magnitude of clavicular elevation. A previous study has shown that this measurement can be used to subdivide the SIS group in two subgroups with specific reaching kinematics [19]. Subjects that increased their clavicular elevation during reaching present significant movement impairments at the trunk, clavicle, and shoulder, while the other subjects present slight movement impairments. The two SIS subgroups were: SIS subjects having clavicular elevation excursion above (SISele; n = 17) and below (SISdep; n = 10) the 95% confidence interval (CI) of the clavicular elevation excursion of the control group. Separate analyses were performed for each SIS subgroup. There were no statistical comparisons between the subgroups.

## Results

The SIS and control groups were similar for age, weight, height, sex and dominance. No significant differences were noted between the two SIS subgroups for age, sex, DASH score and duration of symptoms (Table 1).

### Comparison between control and SIS groups and subgroups at baseline

**Figure 1:**
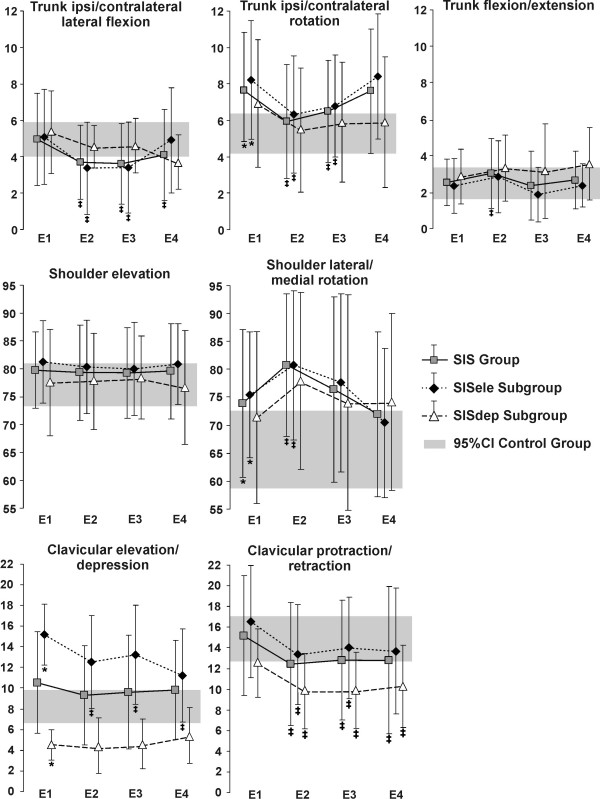
**Mean total joint excursion**. Total joint excursion (mean and standard deviation) before (E1; baseline), during (E2), immediately after (E3) and the day after (E4) movement training with feedback are shown for the SIS group and subgroups (SISele; SISdep). The grey band represents the 95% confidence interval (95%CI) of the control group. * Significant difference at baseline between the group/subgroups with SIS and the control group ^‡ ^Significant difference in the group/subgroups with SIS compared to their baseline trials (E1).

**Figure 2:**
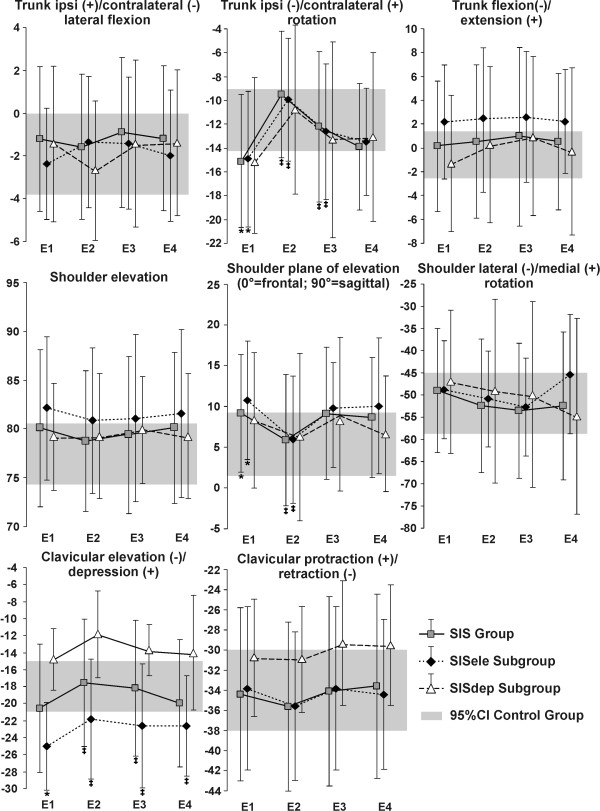
**Mean joint position at the end of reaching**. Joint position at the end of reaching (mean and standard deviation) before (E1; baseline), during (E2), immediately after (E3) and the day after (E4) movement training with feedback are shown for the SIS group and subgroups (SISele; SISdep). The grey band represents the 95% confidence interval (95%CI) of the control group. * Significant difference at baseline between the group/subgroups with SIS and the control group ^‡ ^Significant difference in the group/subgroups with SIS compared to their baseline trials (E1).

Analyses of a previous study [19] have shown that the control group performed reaching using trunk contralateral lateral flexion and ipsilateral rotation, clavicular elevation and retraction, with shoulder elevation and lateral rotation, elbow flexion and wrist extension for the first 50% of movement followed by elbow extension and wrist flexion. The SIS group used the same pattern; however, they used significantly larger trunk rotation (mean difference: 2.6°) and shoulder lateral rotation (9.0°); and finished reaching with the trunk more rotated (4.7°) and with the shoulder in a more anterior plane of elevation (4.1°) (Figures 1 and 2). Compared to the control group, subjects in SISele subgroup performed reaching with greater trunk rotation (3.4°), clavicular elevation (6.3°) and shoulder lateral rotation (11.3°) (Figure 1), as well as less elbow flexion (13.4°). They also finished reaching with more trunk rotation (4.5°) and clavicular elevation (7.1°) and with the shoulder in a more anterior plane of elevation (6.2°) (Figure 2). For their part, the SISdep group used less clavicular elevation (3.5°) (Figure 1). Further detail of these previous analyses can be found in Roy et al. (2008) [19].

### Effect of movement training on pain, reaching speed and EMG activity

**Figure 3:**
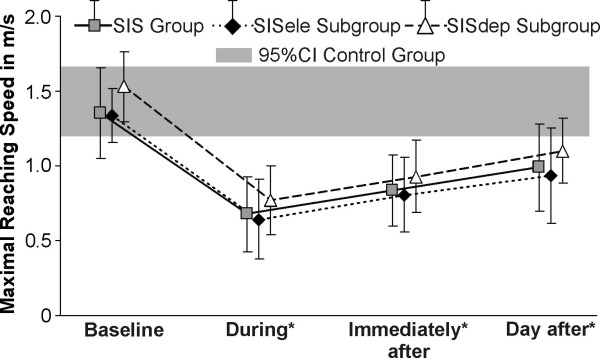
**Maximal hand speed (in m/s) during reaching**. The maximal hand reaching speed (mean and standard deviation) observed before (baseline), during, immediately after and the day after training with feedback is shown. The grey band represents the 95% confidence interval (95%CI) of the control group. * Significant difference in the group/subgroups with SIS compared to their baseline trials (E1).

**Figure 4:**
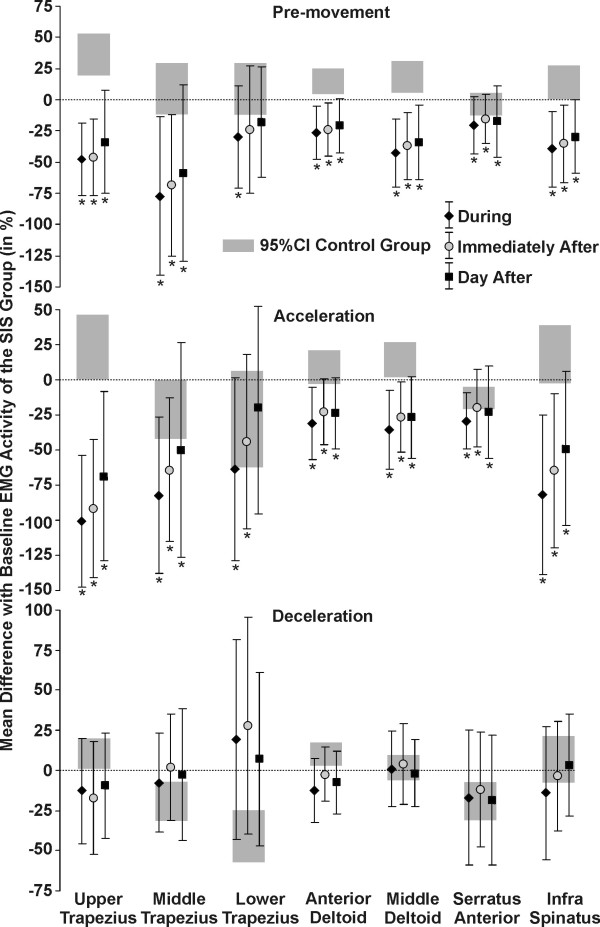
**Mean difference with baseline EMG activity**. The differences (mean and standard deviation) between the EMG activity at baseline and during, immediately after and the day after movement training are shown (0 = no difference with the baseline value) for the SIS group. The differences (mean and standard deviation) at baseline between the EMG activity of the control group and of the SIS group are also plotted (grey band). * Significant difference in the group with SIS compared to their baseline trials.

Compared to their own baseline level, shoulder pain during reaching was significantly reduced in the SIS group and SISele subgroup during (mean difference: 0.3, *P *= 0.02), immediately after (respectively: 0.6 & 0.5, *P *< 0.004) and the day after training (respectively: 0.6 & 0.5, *P *< 0.004), while it remained unchanged in the SISdep subgroup. Maximal reaching speeds were significantly reduced during and following training in the SIS group and in the two SIS subgroups (Figure 3). For the SIS group and subgroups, the EMG activity of all the muscles evaluated, except lower trapezius, was significantly decreased in the pre-movement and acceleration phases during and following training compared to baseline (5.5 to 100.6%) (Figure 4). There were no significant differences during the deceleration phase.

### Effect of movement training on the upper limb kinematic

**Figure 5:**
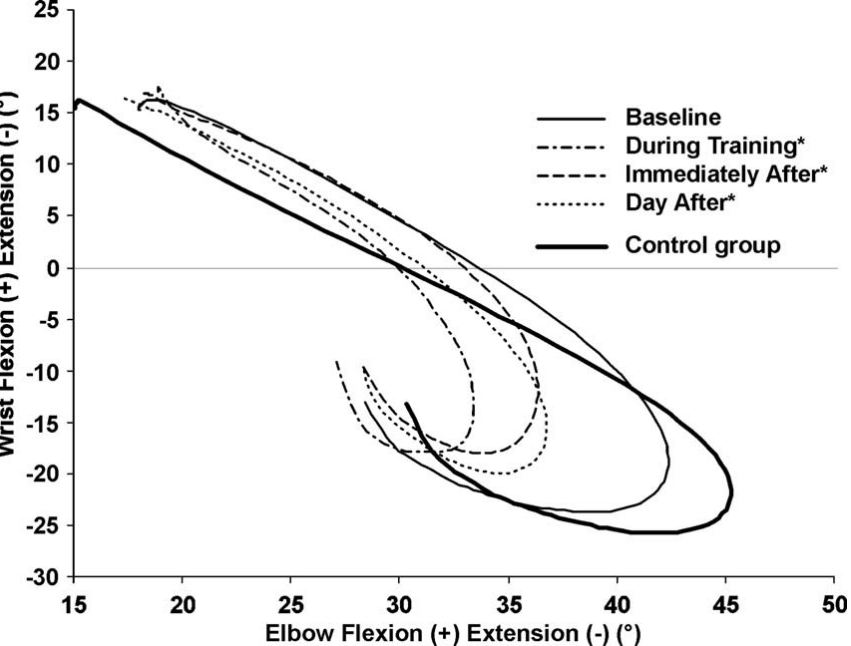
**Interjoint coordination between elbow and wrist flexion/extension**. The interjoint coordination between elbow and wrist flexion/extension observed before (baseline), during, immediately after and the day after movement training are shown for the SIS group. * Significant difference in the group with SIS compared to elbow and wrist excursions at baseline.

Compared to baseline, subjects with SIS used the same pattern of movement during training with feedback. However, excursions of the trunk in lateral flexion (1.2°) and rotation (1.8°), of the clavicle in protraction/retraction (2.7°) and of the elbow (8.7°) and wrist (4.5°) in flexion/extension were significantly decreased, while excursions of the trunk in flexion/extension (0.6°) and of the shoulder in rotation (6.9°) were increased (Figures 1 and 5). In the SISele subgroup, excursion of the trunk in lateral flexion (1.7°) and rotation (1.7°) and of the clavicle in elevation/depression (2.8°) and protraction/retraction (3.3°) were significantly decreased during training compared to baseline, while excursion of the shoulder in rotation was increased (6.5°) (Figures 1 and 5). In the SISdep group, only clavicular protraction/retraction excursion (2.6°) was significantly decreased during training (Figure 1). During training, subjects in the SIS group and in the SISele subgroup also had significantly less trunk rotation (5.6° and 5.3°) and clavicle elevation (3.4° and 3.2°), with the shoulder in a more frontal plane of elevation at the end of reaching (3.5° and 4.3°) (Figure 2).

Immediately following training, excursions of the trunk in lateral flexion (1.3°) and rotation (1.4°), of the clavicle in protraction/retraction (2.3°) and of the elbow (5.3°) and wrist (4.8°) in flexion/extension were still decreased during reaching in the SIS group compared to baseline (Figures 1 and 5). For the subjects in the SISele subgroup, excursion of the trunk in lateral flexion (1.7°) and rotation (1.3°) and of the clavicle in elevation/depression (2.4°) and protraction/retraction (2.5°) were also still significantly decreased (Figure 1). Again, only clavicular protraction/retraction excursion was significantly decreased for the SISdep subgroup (2.6°) (Figure 1). The subjects in the SIS group and in the SISele subgroup also still had significantly less trunk rotation (2.8° and 2.7°) and clavicle elevation (2.5° for both) at the end of reaching immediately following training (Figure 2).

The day after movement training, clavicular elevation/depression excursion (4.0°) and clavicular elevation position at the end of reaching (2.5°) were significantly decreased in the SISele subgroup compared to baseline (Figure 1 and 2). Otherwise, excursion of the trunk in lateral flexion (0.9°), of the clavicle in protraction/retraction (2.3°) and of the elbow (5.8°) and wrist (5.1°) in flexion/extension in the SIS group (Figures 1 and 5), and excursion of the clavicle in protraction/retraction (2.3°) (Figure 1) in the SISdep subgroup were still significantly decreased compared to baseline.

## Discussion

This study is the first to look at the effect of movement training on motor strategies of persons with SIS. Supervised training, aimed at improving individualized movement deficits, was shown to have short-term effects on the upper limb kinematic patterns. For most subjects, these changes were associated with a decrease of shoulder pain during reaching.

Changes observed in the upper limb kinematics during and following training led to some improvements. As observed during the baseline trials, the SIS group used more trunk rotation and finished reaching with the trunk more rotated and the shoulder in a more anterior plane of elevation when compared to healthy subjects. During and immediately following training, all these impairments observed in persons with SIS were reduced. However, these kinematic improvements returned to the baseline level the day after training. According to Doyon and Benali [27], the first steps of learning are characterized by improvement in performance occurring within a single session. Still, the skills are not consolidated and multiple training sessions are needed before their consolidation. Present results support this view with short-term positive changes, but minimal retention 24 hours after. One session, therefore, was not enough to bring permanent changes in motor strategies.

For some joints, movement training led to decreased excursions which increased baseline differences with the control group. These changes could be seen as unfavourable. However, it has been shown that early in motor learning, when persons have to choose how to efficiently manage the various degrees of freedom, control is simplified through the freezing of some degrees of freedom [28]. Such a strategy could be temporarily adopted to facilitate performance by allowing the opportunity to control a few necessary degrees of freedom [28]. As seen in the results (see Figure 5), persons with SIS mostly restricted the movements of the more distal joints with training. By restricting the excursion of the elbow and wrist joints, they could put more emphasis on providing the proper strategy at the shoulder joint to avoid impingement. This adaptation could be seen as an early step in motor learning [28].

EMG activity was decreased during and following training. The baseline differences with the control group were increased for most of the muscles and the standard deviations were larger than the ones of the control group. Previously, it has been shown that the standard deviation is similar between persons with and without SIS [19]. Present findings show that the variability in the muscular performance of the persons with SIS was more important during and following training. According to the learning phases of Fitts & Posner [12], the subjects were involved in the first phase of learning, the cognitive stage. They had to solve the problem first and find out what had to be done to improve shoulder control. This phase required considerable cognitive activity in order to determine the appropriate strategies. Therefore, performances were inconsistent, leading to large within and between subject variability. Such cognitive activities most likely also led to the reduction of reaching speed observed during and following training. This reduction of speed makes it difficult to compare the EMG activity with baseline since lower speed is associated with lower EMG activity [19].

The training session was designed to optimize motor learning. Studies have looked at different ways to enhance training by looking at the best motor learning strategies [9-11,29]. One conclusion of these studies is that subjects have to be actively involved in solving the motor problem during training [9]. The training session was planned around that principle. As a result, to improve the intrinsic error-detection capabilities of the subjects [9]: a) pre-training education with an anatomical model of the shoulder was given [11], and, b) a mirror was used to observe the kinematics of the unimpaired and impaired shoulders in order to give a visual comparison to the subject of the movements that had to be improved. Moreover, to allow active engagement in information processing activities [9,11,29]: a) external feedbacks was given in only half of the trials, and b) subjects had to judge their own performance before the verbal feedback from the physiotherapist. Furthermore, it has been shown that the learning effects are greater when feedback is not given on each trial and when the subjects have to evaluate their performance first [30].

The separate analyses of the two SIS subgroups proved that not all persons with SIS respond the same way to training. Subjects in the SISele subgroup were the ones who seem to have benefited the most from training. With training, they significantly changed their trunk, clavicle and shoulder kinematics leading to a reduction of the baseline differences with the control group. Furthermore, shoulder pain during reaching was significantly reduced. This suggests that, for persons with significant kinematic deficits, rehabilitation of their motor control deficits is an important step for improving their shoulder pain. Indeed, the reduced clavicular elevation could be a sign that they are able to reach without superiorly migrating their humeral head. Thus, these persons most likely reduced the impingement of the subacromial structures leading to the observed decrease of pain. Moreover, by reducing their trunk movement and by moving more into the frontal plane, they demonstrated that they do not prevent their shoulder from moving in a plane where the subacromial space is minimal [22].

In the SISdep subgroup, only clavicular pro/retraction was changed with training, increasing baseline differences with the control group. Furthermore, pain level remained unchanged. It could be argued that training was more relevant for the SISele group. Training was design to correct the specific movement deficits observed in patients with SIS and persons with SIS who use greater clavicular elevation during reaching present the most altered kinematic patterns. Thus, persons presenting mild kinematic deficits may benefit better of other types of rehabilitation, such as strengthening or stretching exercises. However, the small number of subjects in the SISdep subgroup could have contributed to the lack of differences.

Some limitations of this investigation are worthy of note. Only the short-term effect of movement training with feedback was evaluated in this study. Further researches will have to look at its long-term effects. Scapular movement was not evaluated. However, no valid and reliable method was available in our laboratory to characterize scapular dynamic changes. Training with feedback is only one aspect of the rehabilitation program. Evaluation of physical factors, such as muscle strength, endurance and range of motion that could interfere with the ability of persons with SIS to perform arm movement also need to be addressed. Furthermore, it is still unknown, as in other musculoskeletal conditions [31], if the motor control deficits precede or follow the onset of pain and through which cortical or subcortical mechanisms these deficits take place. Only future neurophysiologically based studies will help to better understand the neural mechanisms underlying motor control deficits observed in persons with SIS.

## Conclusion

Movement training with feedback led to short-term changes during arm movements with respect to shoulder pain and upper limb kinematic patterns. Persons with SIS who presented the greater kinematic deficits at baseline were the ones who demonstrated the most significant changes in pain and kinematics following movement training. Thus, rehabilitation strategies should be based on initial kinematic deficits. Our results support the need to evaluate this approach during a long-term training program.

## Abbreviations

ANOVA: Analysis of variance; DASH: Disabilities of the arm, shoulder, and hand; E1: Baseline evaluation; E2: Evaluation during movement training; E3: Evaluation immediately after movement training; E4: Evaluation 24 hours after movement training; SIS: Shoulder impingement syndrome; EMG: Electromyographic

## Competing interests

The authors declare that they have no competing interests.

## Authors' contributions

JSR: participated in the design of the study, carried out the acquisition, the analysis and the interpretation of data and drafted the manuscript.

HM: participated in the design of the study, the analysis and the interpretation of data and drafted the manuscript.

BJM: participated in the design of the study, carried out the acquisition, the interpretation of data and drafted the manuscript.

RL: participated in the development of the study question, enrolled subjects, and participated in the revision of the manuscript.

All authors read and approved the final manuscript.
